# Remodeling of brain morphology in temporal lobe epilepsy

**DOI:** 10.1002/brb3.1825

**Published:** 2020-09-17

**Authors:** Elisabeth Roggenhofer, Sandrine Muller, Emiliano Santarnecchi, Lester Melie‐Garcia, Roland Wiest, Ferath Kherif, Bogdan Draganski

**Affiliations:** ^1^ Neurology Department Department of Clinical Neuroscience HUG University Hospitals and Faculty of Medicine Geneva Geneva Switzerland; ^2^ Department of Clinical Neurosciences LREN CHUV University of Lausanne Lausanne Switzerland; ^3^ Berenson‐Allen Center for Non‐Invasive Brain Stimulation Cognitive Neurology Department Beth Israel Medical Center Harvard Medical School Boston MA USA; ^4^ Siena Brain Investigation and Neuromodulation Lab Department of Medicine, Surgery and Neuroscience University of Siena Siena Italy; ^5^ Applied Signal Processing Group Swiss Federal Institute of Technology Lausanne (EPFL) Lausanne Switzerland; ^6^ Support Center for Advanced Neuroimaging Institute for Diagnostic and Interventional Neuroradiology University Hospital Inselspital University of Bern Bern Switzerland; ^7^ Department of Neurology Max‐Planck‐Institute for Human Cognitive and Brain Sciences Max Planck Society Leipzig Germany

**Keywords:** Bayesian model selection, BMS, computational anatomy, hippocampus, magnetic resonance imaging, multivariate Bayesian modeling, MVB, temporal lobe epilepsy

## Abstract

**Background:**

Mesial temporal lobe epilepsy (TLE) is one of the most widespread neurological network disorders. Computational anatomy MRI studies demonstrate a robust pattern of cortical volume loss. Most statistical analyses provide information about localization of significant focal differences in a segregationist way. Multivariate Bayesian modeling provides a framework allowing inferences about inter‐regional dependencies. We adopt this approach to answer following questions: Which structures within a pattern of dynamic epilepsy‐associated brain anatomy reorganization best predict TLE pathology. Do these structures differ between TLE subtypes?

**Methods:**

We acquire clinical and MRI data from TLE patients with and without hippocampus sclerosis (*n* = 128) additional to healthy volunteers (*n* = 120). MRI data were analyzed in the computational anatomy framework of SPM12 using classical mass‐univariate analysis followed by multivariate Bayesian modeling.

**Results:**

After obtaining TLE‐associated brain anatomy pattern, we estimate predictive power for disease and TLE subtypes using Bayesian model selection and comparison. We show that ipsilateral para‐/hippocampal regions contribute most to disease‐related differences between TLE and healthy controls independent of TLE laterality and subtype. Prefrontal cortical changes are more discriminative for left‐sided TLE, whereas thalamus and temporal pole for right‐sided TLE. The presence of hippocampus sclerosis was linked to stronger involvement of thalamus and temporal lobe regions; frontoparietal involvement was predominant in absence of sclerosis.

**Conclusions:**

Our topology inferences on brain anatomy demonstrate a differential contribution of structures within limbic and extralimbic circuits linked to main effects of TLE and hippocampal sclerosis. We interpret our results as evidence for TLE‐related spatial modulation of anatomical networks.

## Graphical abstract

In focal cryptogenic epilepsy, morphological abnormalities often remain undetected at the macroscopic brain level. We use Multivariate Bayesian modeling analysis to link the spatial patterns of brain anatomy remodeling in temporal lobe epilepsy patients with their clinical phenotype. This approach allows detecting the regions which have an impact on the ongoing disease process. Our study could serve as proof‐of‐concept for the added value of topology analysis for detection of focal structural abnormalities in focal epilepsy that can be further implemented for other brain disorders.

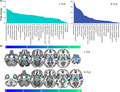

## 1 INTRODUCTION

Temporal lobe epilepsy (TLE), one of the most common forms of focal epilepsy, is associated with progressive cognitive dysfunction and resistance to antiepileptic drug therapy (Wiebe & Jette, 2012). Although considered for many years as disorder related to focal temporal lobe pathology, there is strong evidence for disruptions in a widespread cortico‐subcortical network (Bernhardt, Hong, Bernasconi, & Bernasconi, 2013; Bonilha et al., 2013; Concha, Kim, Bernasconi, Bernhardt, & Bernasconi, 2012). Given the fact that the TLE clinical phenotype is modified as function of disease progression, there is clear need to shed light on spatial and temporal dynamics of changes within affected brain circuits.

Computational anatomy studies in TLE, using in vivo brain magnetic resonance imaging (MRI), demonstrate a specific pattern of cortical volume loss and changes in corresponding white matter pathways that extend beyond mesial temporal lobe structures (Bernhardt, Hong, et al., 2013; Keller & Roberts, 2008). Theoretical work and studies on animal models suggest that TLE‐associated network remodeling follows a specific temporal trajectory (Leite et al., 2005; Sutula, 2004). Recent report provided empirical evidence for the assumption of bidirectional brain anatomy changes during disease progression with initial seizure‐dependent boost in neurogenesis followed by gliosis due to depletion of hippocampal stem cells and shift toward astrocytes production (Sierra, Grohn, & Pitkanen, 2015; Sierra, Martin‐Suarez, et al., 2015). In humans, cross‐sectional (Bonilha et al., 2004) and longitudinal studies (Bernhardt, Kim, & Bernasconi, 2013) corroborated continuous changes affecting hippocampal volume loss in chronic TLE stages. Unpublished findings from our own group confirmed the notion of bidirectional hippocampus alterations and demonstrated hippocampus volume increase in early stages of TLE, followed by progressive atrophy of the hippocampus ipsilateral to seizure onset (Roggenhofer et al., 2019). The assumption of differential temporal dynamics of brain anatomy changes within the TLE network remains to be tested, particularly in relationship with individual clinical phenotype.

Compared to investigation of temporal trajectories of TLE‐induced brain circuit changes, our knowledge in the spatial domain of network modulation remains very limited. Previous studies demonstrated strong links between clinical phenotype and functional brain network organization in the case of left‐ or right‐lateralized TLE (Doucet, Osipowicz, Sharan, Sperling, & Tracy, 2013), however, comparable work in the field of brain anatomy lacks specificity. Current statistical analyses in the framework of computational anatomy describe the spatial pattern of TLE pathology without formally testing the question about interdependencies between spatially segregated structural findings. At present, it remains unclear to which degree a particular structure within the TLE specific network is involved in underlying pathological mechanisms and how these regional interdependencies evolve over time.

Main goal of our study is to investigate differential topology of brain anatomy changes within TLE circuits. We hypothesize that the ipsilateral hippocampus is the structure with strongest contribution to TLE‐induced brain anatomy remodeling. Additionally, we predict that limbic and extralimbic structures with weaker contribution to TLE anatomical pattern show differential involvement depending on the laterality of seizure onset and presence or absence of MRI‐visible hippocampus sclerosis. To test the added value of multivariate analysis over classical mass‐univariate analysis, we use the same dataset of TLE patients and healthy controls reported in our previous study (Roggenhofer et al., 2019). Brain anatomy feature extraction is performed in the established framework of voxel‐based morphometry (VBM), followed by multivariate Bayesian (MVB) statistics adapted for use of structural MRI data.

## 2 METHODS

### 2.1 Participants

For statistical analysis, we used cross‐sectional data from 128 TLE patients (69 females, mean age ± standard deviation: 38.17 ± 10.81 years, age range: 19–63 years) and 120 sex‐ and age‐matched healthy volunteers (63 females, mean age ± standard deviation: 36.01 ± 9.89 years, age range: 17–60 years; Table 1) that were reported previously (Roggenhofer et al., 2019). The protocol was approved by the local Ethics Committee. Informed consent was obtained from each participant. All procedures were performed in accordance with national and international guidelines.

**Table 1:** Demographic and clinical information of study participants

	No.	Gender [no.]		Age [year]	Drug resistance [no.]		AO [year]	TD [year]	TIV [l]
		F	M		D+	D−			
TLE	128	69	59	38 ± 11	52	76	19.2 ± 14.3	19.0 ± 13.7	1.40 ± 0.17*
L TLE	72	45	27	39 ± 11	36	36	20.4 ± 15.5	18.2 ± 13.3	1.38 ± 0.15*
R TLE	56	24	32	38 ± 11	40	16	17.7 ± 12.8	20.0.±13.5	1.43 ± 0.19
MTS	57	34	23	40 ± 10	7*	50*	15.6 ± 13.2*	24.2 ± 13.3*	1.36 ± 0.18*
MRI−	71	35	36	37 ± 11	45*	26*	22.1 ± 14.5*	14.8 ± 11.7*	1.43 ± 0.16*
C	120	63	57	36 ± 10	–	–	–	–	1.45 ± 0.14*
Total	248	132	116	37 ± 10					1.42 ± 0.16

Note

Statistical significance for group differences between TLE (sub)group and C or between MTS and MRI− TLE of *p* < .05 marked by *.

Abbreviations: AO, age of disease onset; C, healthy control volunteers; D−, drug‐resistant; D+, drug‐responsive; f, female; L TLE, left lateralized; m, male; MRI−, MRI negative, without macroscopic MRI brain changes; MTS, mesial temporal lobe sclerosis; R TLE, right‐lateralized TLE; TD, time duration of disease; TIV, total intracranial volume; TLE, temporal lobe epilepsy.

The diagnosis of TLE followed the well‐established criteria of the International League Against Epilepsy (Berg et al., 2010; Engel, 2006) including (a) clinical aspects of seizures like semiology, onset, and history, (b) standard and/or sleep electroencephalography with or without hyperventilation and intermittent photic stimulation additional to long‐term video‐electroencephalography (King et al., 1984) monitoring, and (c) neuro‐radiological assessment. The evaluation of lateralization of the epileptogenic seizure onset zone, that is, what hemisphere was affected, depended on seizure semiology, evidence of unilateral epileptic activity in serial routine or long‐term video‐EEG monitoring and MRI findings. Patients without strong evidence for lateralization, bilateral, lateral temporal or extra‐temporal foci or with macroscopically evident brain pathology outside the mesial temporal lobe were excluded from subsequent analysis. Additional exclusion criteria included history of psychogenic nonepileptic seizures, autoimmune etiology, history of alcohol or drug abuse, history of brain trauma and evidence of ischemic, hemorrhagic brain lesions or tumors.

Among the 128 patients, 56 had right‐lateralized TLE and 72 left‐lateralized TLE (Table 1). The neuro‐radiological assessment confirmed the absence of macroscopic anatomical abnormalities in a total of 71 MRI negative patients (MRI−), whereas the remaining 57 patients were diagnosed with MTS (Table 1). 76 of 128 patients had a pharmaco‐resistant type of epilepsy. The mean age of epilepsy onset was 19.2 ± 14.3 years. The disease duration was averaged at 19.0 ± 13.7 years. The total intracranial volume was higher for healthy controls as for patients with TLE (Table 1).

### 2.2 Magnetic resonance imaging and data processing

The MRI protocol consisted of T1‐weighted images acquired on a 1.5 Tesla Philips INTERA system (Philips Medical Systems) using a 3D magnetization prepared rapid gradient echo protocol (MP‐RAGE) yielding 150 contiguous slices (TE = 4.6 ms, TR = 30 ms, flip angle = 30°, FOV = 250 mm, matrix 256 × 256, voxel size 1 mm^3^ isotropic).

Image preprocessing was performed using the SPM12 software package (Statistical Parametric Mapping, www.fil.ion.ucl.ac.uk/spm) running under Matlab 7.13 (Mathworks Inc.). The algorithm followed the default settings including automated tissue classification in the “unified segmentation” framework (Ashburner & Friston, 2005) using a novel set of brain tissue priors showing increased accuracy for subcortical structures (Lorio et al., 2016). Following this step, gray and white matter probability maps were spatially registered to a standardized Montreal Neurological Institute space using the diffeomorphic algorithm based on exponentiated lie algebra—DARTEL (Ashburner, 2007). The resulting gray matter probability maps were scaled with the corresponding Jacobian determinants to preserve the initial total amount of signal intensity followed by spatial smoothing using an isotropic Gaussian kernel of 8 mm full‐width‐at‐half‐maximum.

### 2.3 Multivariate Bayesian analysis

The MVB statistics followed a mass‐univariate analysis investigating the temporal dynamics of structural remodeling in TLE (Roggenhofer et al., 2019). We used whole‐brain two‐sample *t* tests on gray matter volume maps to demonstrate a pattern of volume differences and their modulation by side of seizure onset additional to presence or absence of hippocampal sclerosis. Here, we combined the significant clusters of the classical VBM approach separately for right and left laterality followed by binarization of the resulting pattern. We used atlas information (probabilistic and maximum probability tissue labels—derived from the “MICCAI 2012 Grand Challenge and Workshop on Multi‐Atlas Labeling” www.masi.vuse.vanderbilt.edu/workshop2012) to label anatomically distinct areas within the binarized VBM pattern. The anatomical labels as region‐of‐interest (ROI) provided the spatial constraints for multivariate decoding and Bayesian model selection.

Multivariate Bayesian was implemented to decode disease‐related patterns from structural brain images (Friston et al., 2008). This decoding framework relies on a model inversion using a variational Bayesian implementation of expectation maximization, to furnish the model evidence and the conditional density of the model's hyperparameters (Friston & Kiebel, 2009). Free energy is an information theory quantity derived from physics that bounds the log evidence of a model of data (Friston, 2009). The multivariate model allows drawing inferences about where and how TLE pathology is represented in the brain by evaluating competing anatomical coding hypotheses on a group level. Therefore, MVB provides the statistical evidence for each regional model that the structural data predict the pathology (Kherif & Muller, 2020). We emphasize that the objective of this MVB approach is not to predict the pre‐ or absence of epilepsy itself as the diagnosis is a known parameter but to enable inferences on distinct regional models and differentiate the individual degree of contribution to a pattern of dynamic epilepsy‐associated brain remodeling.

We used 6 different designs as MVB models including two groups—healthy volunteers and a subgroup of TLE patients. The subgroups were defined based on clinical phenotype—laterality of seizure onset (left and right TLE) and presence (MTS) or absence (MRI−) of temporal lobe pathology. Bayesian model comparison implies the prior generation of multiple regional hypotheses (i.e. ROI or models) to be compared. As input for each Bayesian model, we use the voxel information based on gray matter probability maps within anatomically distinct atlas‐defined ROI (see above “Magnetic resonance imaging and data processing”). The calculation of the model evidence permits Bayesian Model Comparison and selection (Penny, Flandin, & Trujillo‐Barreto, 2007). Bayesian Model Selection (Penny et al., 2007; Stephan, Penny, Daunizeau, Moran, & Friston, 2009) is applied on the created models to compare different spatial hypotheses (Hulme, Skov, Chadwick, Siebner, & Ramsoy, 2014) using both, the model log evidence (Free Energy) and the parameter densities. Regional models maximizing free energy are more likely to confirm the model‐specific prediction of TLE disease and subtype pathology. Statically significant clusters at the group level were identified using the classical SPM mass‐univariate approach. Each cluster was labeled according to their ROI.

**FIGURE 1:**
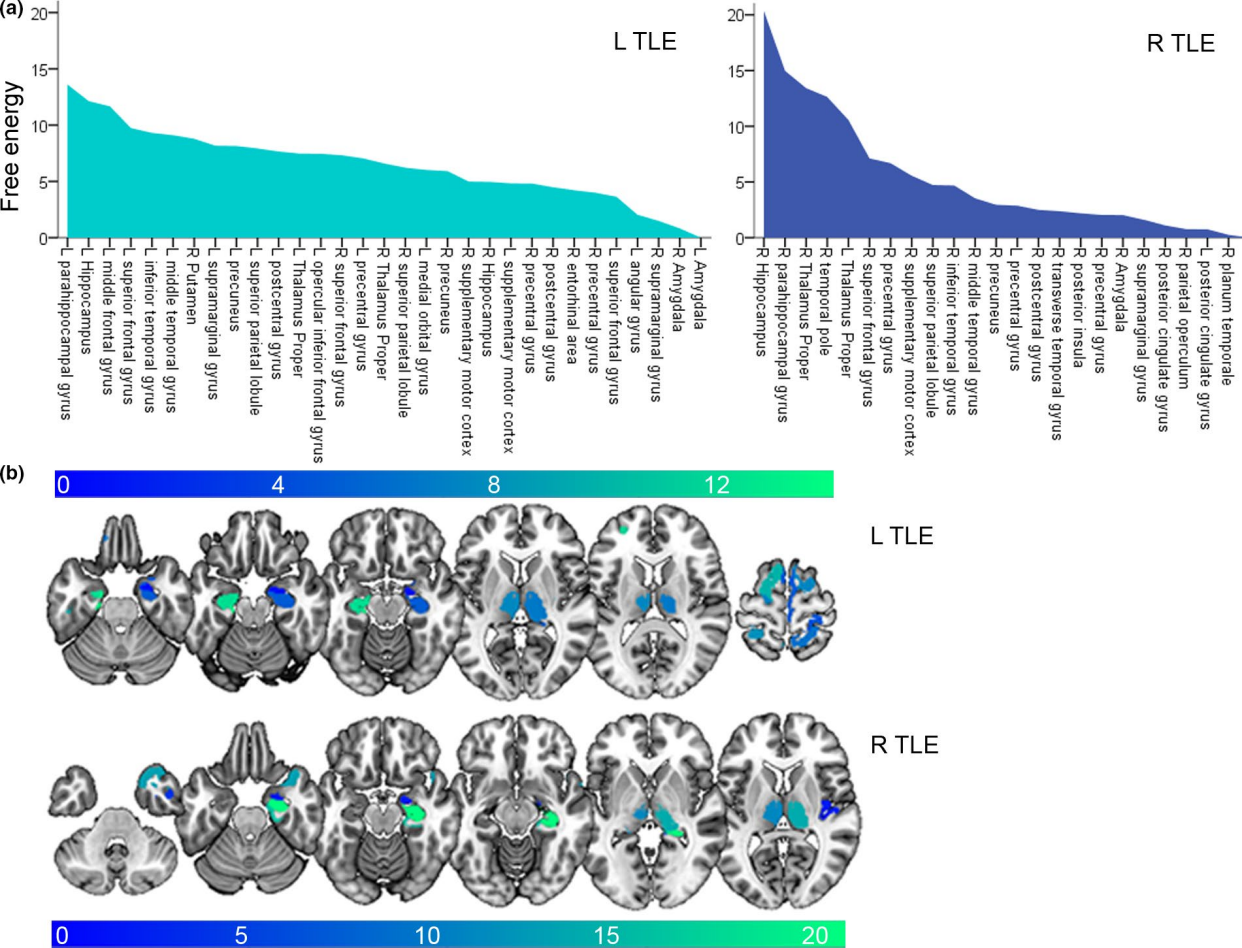
Structural remodeling in lateralized TLE patterns: main effects of disease. Multivariate Bayesian modeling (MVB) based on differences in volume estimates between healthy control volunteers and L or R TLE. Free energy values plotted for each parcellated region‐of‐interest (ROI) displaying patterns of structural remodeling in L and R TLE based on sparse spatial prior. (a) Values for each ROI shown in descending order and (b) projected to standard MNI space for cortical ROIs. L TLE, left lateralized; R TLE, right‐lateralized TLE

We quantify the contribution of regions to the presence of diagnosis within the pattern of structural brain reorganization and ranked regions dependent on their free energy values. Rankings were performed in left and right TLE in comparison with healthy volunteers (Figure 1a) and, respectively, in left and right MTS and MRI− TLE (Figure 3b). To provide proportional comparability, free energy values of subgroups were scaled to subgroup‐dependent maxima (Figure 3a). Statistical ANOVA analysis revealed differences of free energy values between TLE subtypes within the same TLE laterality. Left‐ and right‐lateralized TLE parameters cannot be directly matched due to different selections of ROIs.

### 2.4 Statistical analysis

**FIGURE 2:**
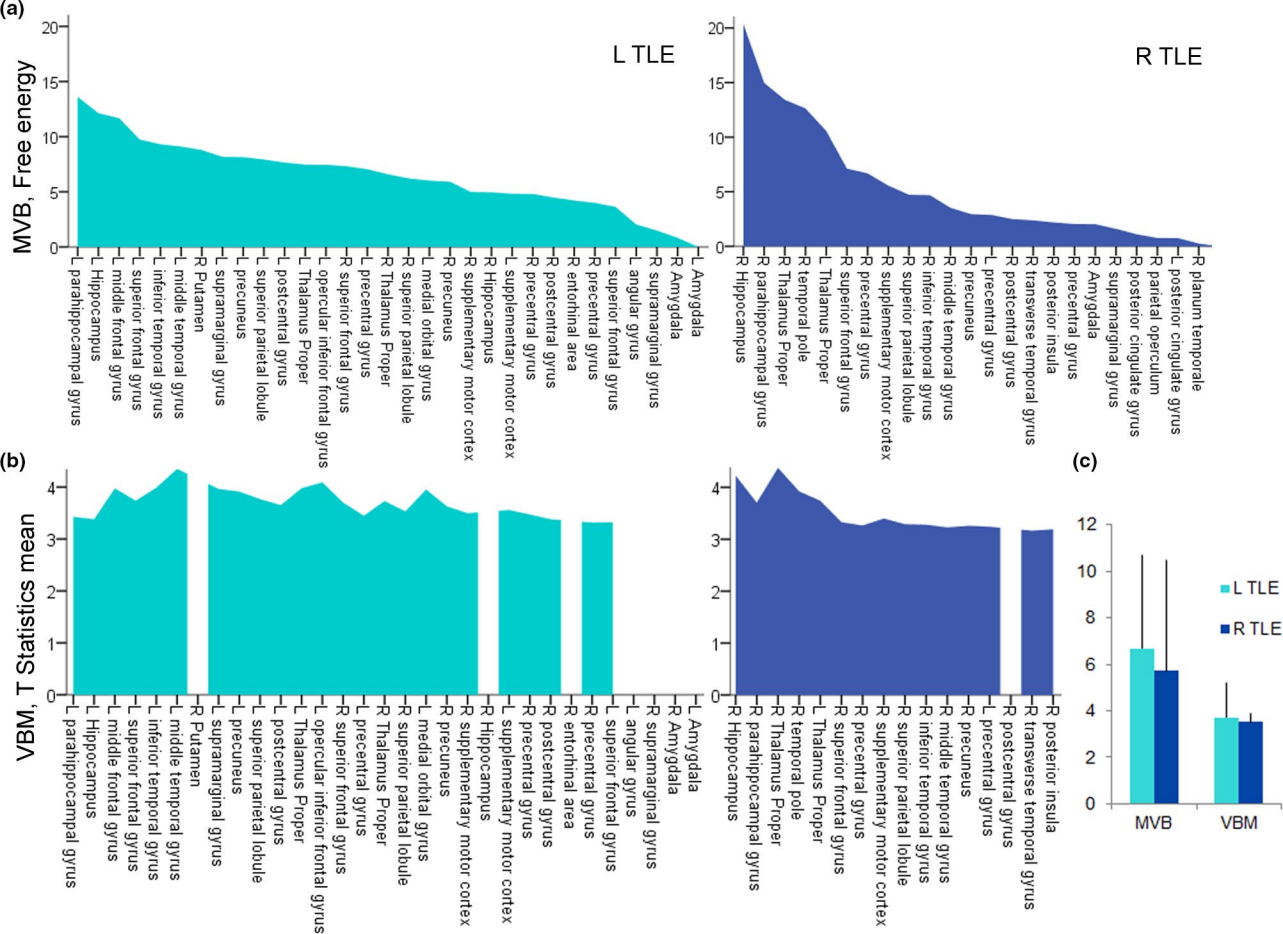
Comparison between uni‐ and multivariate method. Bar plots represent ROI‐dependent results based on MVB contrasted with VBM and differences in volume estimates between healthy volunteers and L or R TLE. In both diagrams, values rank in descending order of free energy. Missing structures in VBM rankings indicate T values below statistical threshold. (a) Free energy plotted across parcellated regions involved in structural remodeling for L and R TLE. (b) Means of T‐statistics plotted across parcellated regions for L and R TLE, statistical threshold of *p* < .001, uncorrected. (c) Free energy values for MVB contrasted with T‐statistics for VBM, averaged across ROIs for L and R TLE, plotted in A and B (mean ± standard deviation). L TLE, left lateralized, MVB, multivariate Bayesian, R TLE, right‐lateralized TLE, VBM, voxel‐based morphometry.

Finally, we compared rankings based on multivariate pattern modeling to the established VBM approaches. For detecting group differences between left‐ or right‐lateralized TLE and healthy volunteers, we applied a statistical threshold at *p* < .001, uncorrected. VBM rankings depended on statistical parametric mapping and T‐map values, averaged across distinct anatomical structures, that is, ROIs or regional models. For visualization of T‐statistics, we adopted the same ranking sequences like for MVB results (Figure 2). The multivariate statistics (model log evidences) and the mass‐univariate statistics (averaged T‐statistics) cannot be compared for the same ROI. Instead, we propose to compare the overall ranking of the ROIs.

In a first analysis, we computed the MVB models using four types of structural abnormalities representations: compact, sparse, smooth, and support representations for each parcellated ROI (Friston et al., 2008). The model log evidences were compared with the same ROIs across different kernels. Models using a sparse representation were the most predictive ones for both individual mesial temporal lobe structures and the mean of gray matter ROIs (data not shown).

Weights attributed to each voxel in ROIs suggest sparse structural coding. Therefore, sparse priors were uniformly chosen in this study to optimize the implemented models. Results are illustrated for left‐lateralized TLE on the left side and for right‐lateralized TLE on the right side on all following figures.

## 3 RESULTS

### 3.1 Main effects of disease

Using VBM, we defined the TLE‐associated pattern and labeled the involved distinct anatomical structures. Based on MVB, we calculated free‐energy parameters for each of these structures.

The topological MVB ranking determines that volume estimates in ipsilateral mesial temporal lobe regions most contribute to TLE (Figure 1a, b). The two most contributing regions cover the ipsilateral hippocampus and para‐hippocampal gyrus independent of laterality. The consequent ranking positions are located prefrontally in superior and middle frontal gyrus for left TLE and bilaterally thalamic and ipsilateral temporal polar for right TLE. In left TLE, higher free energy values are achieved for ipsilateral high frontal and parietal regions including superior frontal, postcentral gyrus, superior parietal lobule, precuneus, and supramarginal gyrus as well as lateral temporal regions including middle and inferior temporal gyrus. In contrast, the ipsilateral hippocampus and bilateral thalamus are ranked higher in right TLE.

In left TLE, the affected pattern differs in comparison to right TLE. The left‐sided pattern is characterized by a lower maximal free energy value of 13 in left TLE in comparison with 20 in right TLE (Figure 1a), higher mean values (6.7 ± 4.0 in left TLE and 5.7 ± 4.7 in right TLE), and higher numbers of affected structures in general: *n* = 30 ipsilateral and *n* = 13 contralateral in left TLE and *n* = 24 and 3 in right TLE (Figure 1a). The range between minimal and maximal values is more restrictive for left TLE (0.8; 13.6) compared with right TLE (0.3; 20.4).

### 3.2 Comparison between mass uni‐ and multivariate methods

To contrast the method with established volumetric morphology approaches, we paralleled the MVB‐based ranking with a ranking based on VBM ROI‐dependent means of T‐statistics. For the whole remodeling pattern, the free energy values demonstrate a higher variance between distinct ROIs based on the MVB approach (6.7 ± 4.0 in left TLE and 5.7 ± 4.7 in right TLE) compared with VBM‐based T‐statistics (3.7 ± 1.5 in left TLE and 3.5 ± 0.4 in right TLE) (Figure 2).

In terms of VBM ranking positions, we achieve highest scores for the ipsilateral thalamus, hippocampus, and temporal pole in right TLE. In right TLE, rankings provide highly congruent results based on the two distinct methods. Ranking positions of thalamic and para/hippocampal structures are switched between both methods. The VBM approach estimates an increased involvement of the ipsilateral thalamus and inversely downgrades hippocampal and para‐hippocampal areas. In left TLE, VBM rankings differ extensively from MVB‐based ones in diverse positions including top ranks. The first five VBM ranking positions comprise ipsilateral lateral temporal structures implying the middle and inferior temporal gyrus and prefrontal structures implying the opercular inferior frontal and middle frontal gyrus next to the thalamus. The VBM ranking compared with the MVB approach supports ipsilateral lateral temporal structures (middle temporal gyrus), the fronto‐basal cortex (inferior frontal gyrus and medial orbital gyrus), and the bilateral thalamus to a higher degree. Structures dominating the MVB‐based ranking compared with VBM are mesial temporal lobe structures implying the para‐hippocampal and bilateral hippocampal regions and the high frontal regions including the ipsilateral pre‐, postcentral gyrus, and bilateral superior frontal gyri.

### 3.3 Structural remodeling in TLE subtypes

**FIGURE 3:**
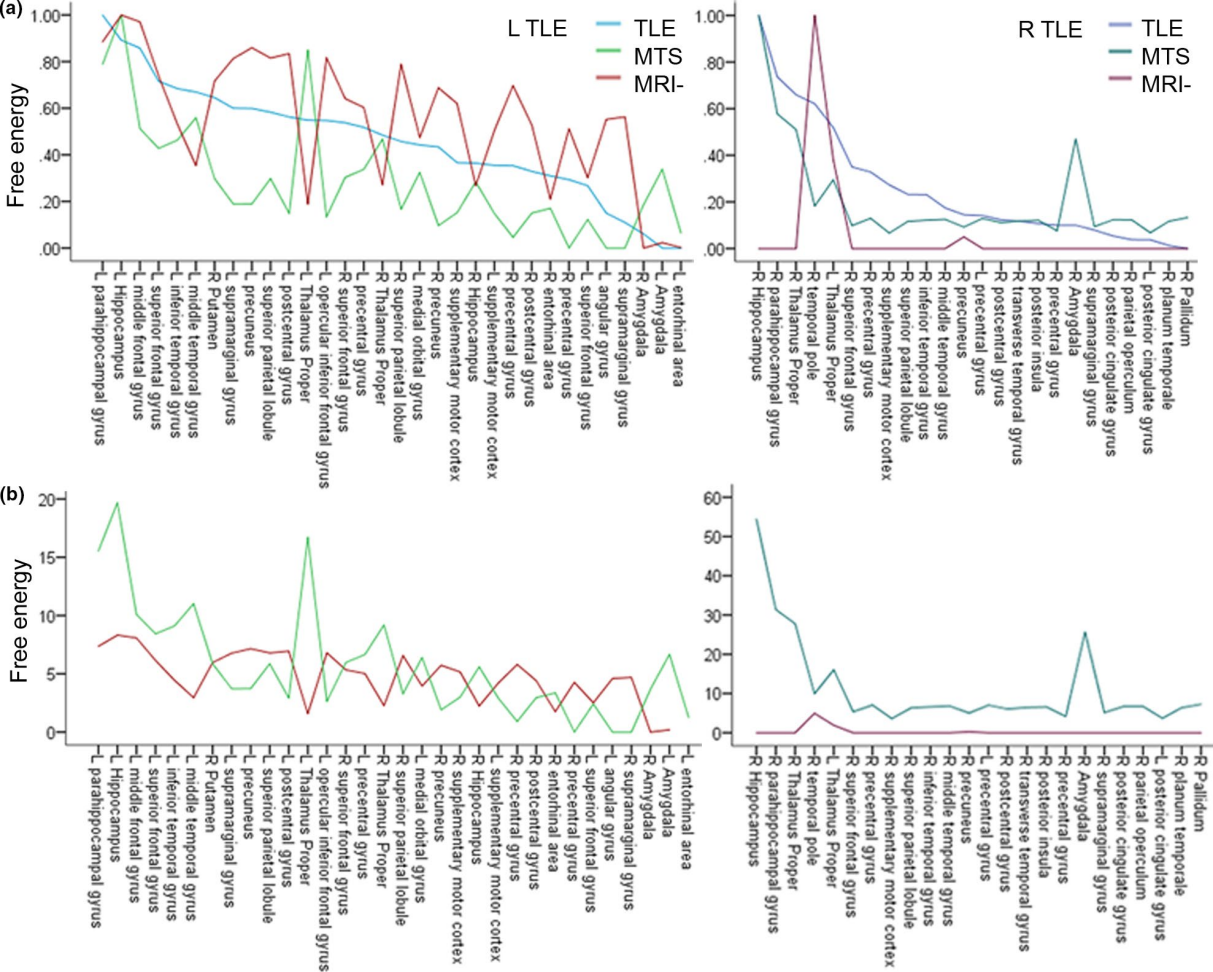
Structural remodeling in TLE subtypes. MVB based on differences in volume estimates between healthy volunteers and left and right MTS and MRI− TLE. Free energy values plotted across parcellated regions involved in structural remodeling. Values are shown in descending order of free energy and based on sparse spatial prior. (a) Line diagram displays free energy values normalized to maximum of each TLE subgroup. (b) Absolute values of free energy are plotted for MTS and MRI− subgroups. L TLE, left lateralized; MRI−, MRI negative, without macroscopic MRI brain changes; MTS, mesial temporal lobe sclerosis; R TLE, right‐lateralized TLE

To evaluate regional specificity between TLE subtypes, MVB models were established for MTS and MRI− subtypes of TLE. Structural changes in MTS compared to MRI− and in right lateralized compared to a left‐lateralized epilepsy patterns (R MTS > R MRI−, *p* = .0002 and R MTS > R TLE, *p* = .030; Figure 3a, b) contribute most to structural remodeling, measured by higher absolute values of free energy. Patterns of structural remodeling overlap between right TLE with MTS and TLE without subdifferentiation. MTS TLE differs in a proportionally stronger involvement of the ipsilateral amygdala and to a lower degree of the contralateral thalamus (Figure 3). Structural differences in right MRI− TLE do not contribute for differentiation except the ipsilateral temporal pole, contralateral thalamus, and ipsilateral precuneus.

In left TLE, extrahippocampal patterns of structural remodeling differ between the whole TLE cohort and distinct TLE subtypes. Both subtypes, left MTS and MRI−, exhibit the ipsilateral hippocampus as the mayor structure decisive for the disease (Figure 3 left). In consequent positions in MTS TLE, bilateral thalami as well as mesial and lateral temporal lobe regions including the para‐hippocampal, middle, and inferior temporal gyrus next to the amygdala precede the ranking, followed by medial prefrontal areas like the middle and superior frontal gyrus. In MRI− TLE, prefrontal next to parietal regions dominate the mayor ranking positions including the ipsilateral middle and inferior frontal, the postcentral and supramarginal gyrus, the precuneus and bilateral superior parietal lobules. Comparing remodeling patterns directly between left MTS and MRI− TLE, bilateral amygdala, thalamic and entorhinal regions almost exclusively contribute for differentiation MTS. In MRI− TLE, prefrontal and parietal regions are predominantly important including the inferior frontal gyrus, supplementary motor area, pre‐ and postcentral gyrus, precuneus, angular, and supramarginal gyrus.

## 4 DISCUSSION

Our study provides unique empirical evidence for a differential contribution of brain regions to the process of brain anatomy remodeling in TLE. Using multivariate statistical methods allowing for topology inferences, we quantify the individual contribution of structures within limbic and extralimbic circuits to the TLE‐associated brain anatomy pattern. We identify the ipsilateral hippocampal complex as main driver of spatial dynamics in TLE, whereas thalamus and a number of cortical areas show differential contribution depending on the laterality of the epileptogenic focus and mesial temporal lobe pathology. We interpret our findings as correlates of differential spatial trajectories within the TLE network that are also subjected to changes in due course of disease. The translation of our approach to clinical usage could provide a novel in vivo diagnostic tool for noninvasive localization of an epileptogenic focus, not detectable by conventional radiological assessment.

### 4.1 Main effects of disease

Our main finding is that the hippocampal complex is the most discriminative region for TLE‐related remodeling of brain anatomy. Distinct morphology inferences dependent on hippocampal sclerosis corroborate that the hippocampal topology plays a decisive role in TLE‐related spatial modulation of anatomical networks, supplementary to the previously described temporal dynamics. We observed a common pattern for TLE patients with mesial temporal lobe pathology that includes the ipsilateral hippocampus, para‐hippocampal gyrus, amygdala, and bilateral thalamus—all structures representative of the brain anatomy network implied in TLE (Maccotta, Moseley, Benzinger, & Hogan, 2015; Mathern, Babb, Vickrey, Melendez, & Pretorius, 1995). Given the role of thalamus in the seizure propagation across both hemispheres (Blume, 2009), we interpret our bilateral thalamic findings as confirmation for its important role in the initiation (Li et al., 2014; Toyoda, Bower, Leyva, & Buckmaster, 2013), modulation, and propagation (Barron, Fox, Laird, Robinson, & Fox, 2012; Bertram, Mangan, Zhang, Scott, & Williamson, 2001; Norden & Blumenfeld, 2002) of seizures.

Laterality of the seizure onset influences the spatial pattern in such way that frontoparietal and latero‐temporal structures have a predominant role in left TLE compared with right TLE, a result corroborated by VBM (Riederer et al., 2008) and cortical thickness studies (Kemmotsu et al., 2011). Further, we demonstrate a decentralized, but less severely affected pattern in left TLE. These findings are supported by previous reports where patients with left TLE showed more widespread and diffuse abnormalities including cortical volume loss (Kemmotsu et al., 2011; Riederer et al., 2008) and changes in white matter fiber tracts (Ahmadi et al., 2009) extending to contralateral regions.

The asymmetry in topology patterns could be a consequence of hemisphere‐specific rates of brain fiber tract maturation. Quantitative and diffusion imaging corroborated that maturation occurs earlier and evolves quicker in the left than in the right hemisphere (O'Muircheartaigh et al., 2013) whereas global and local efficiencies are significantly decreased until early adulthood (Zhong, He, Shu, & Gong, 2016). In the context of quicker left‐hemispheric development and less integrated connection with less efficient communication, the left hemisphere tends to be more susceptible to initial events like febrile convulsions and early onset seizures (Kemmotsu et al., 2011). Analogously, hippocampal sclerosis is observed more often in left rather than right hemisphere after febrile convulsions (Janszky et al., 2003). Correspondingly, age‐related maturation of white matter is delayed in children with new‐onset epilepsy (Chiron et al., 1997; Hutchinson et al., 2010). In our dataset, the age at the first seizure was not different between left‐ and right‐lateralized TLE favoring the argumentation that rates of maturation differ across hemispheres. Furthermore, left hemispheric white matter connectivity in individuals with left‐sided language dominance exhibit a more widespread pattern and comprises more complex hippocampal connections (Powell et al., 2007). This can provide a physiological network basis for a more diffuse and extensive left‐lateralized seizure propagation underlying network deterioration.

### 4.2 Differential regional contribution in TLE subtypes

Another important finding is the evidence for differential contribution of specific brain structures to the TLE pattern under the modulatory impact of presence or absence of mesial temporal lobe pathology. The ipsilateral hippocampus volume is highly contributory to group differences between healthy controls and all TLE subtypes except for right MRI− patients where thalamus, temporal polar cortex, and insula achieved highest ranking. Our results, showing a principal difference of regional contribution to brain remodeling depending on the presence or absence of mesial temporal lobe sclerosis, confirm the supposition that these represent the same nosological entity or lie along a biologic continuum (Labate, Cerasa, Gambardella, Aguglia, & Quattrone, 2008; Mumoli et al., 2013). We go further to claim that the evidence for differential regional contribution adds another layer of complexity linked to time‐dependent modulation of brain structure in TLE. This is in line with our previous findings of a switch from hippocampus volume increases to decreases that could be interpreted as turning point with impact on remote regions within the TLE‐affected network (Roggenhofer et al., 2019).

The regions, decisive for TLE in absence of mesial temporal lobe pathology MRI−, represent a widespread distributed pattern including motor, premotor, and associative areas. Our findings corroborate previous reports demonstrating either absence of brain anatomy changes (Alhusaini et al., 2013; Coan, Campos, Beltramini, et al., 2014; Mueller et al., 2006; Peng et al., 2014) or implication of thalamus and sensorimotor cortex (Coan, Campos, Yasuda, et al., 2014; Labate et al., 2008; Mueller et al., 2009; Riederer et al., 2008). Preceding studies in MRI− TLE support the involvement of frontal and parietal cortices, especially the sensorimotor cortex mainly due to excitotoxicity induced neuronal loss (McDonald et al., 2008; Mueller et al., 2009).

### 4.3 Methodological considerations

The descriptive comparison demonstrates the significant advantage in discriminative power when using multivariate instead of univariate methods to answer the question about differential regional contribution to brain remodeling in TLE. The MVB findings, confirming the hippocampus as main target of spatial remodeling, are in line with previous computational anatomy studies using univariate statistics (Bernhardt et al., 2008; Moran, Lemieux, Kitchen, Fish, & Shorvon, 2001).

The VBM analysis downgraded the role of the mesial temporal lobe structures in both right and left TLE and increased rankings in favor of cortical and subcortical structures known to be secondarily implicated in seizure. Indeed, one can infer generic disease‐associated structural differences by established VBM approaches. But the method does not allow drawing causal inferences about inter‐regional dependencies in brain anatomy. Disseminated patterns of structural reorganization are less likely to be identified in VBM approaches and inter‐regional dependencies are not considered. In particular, mesial temporal lobe regions vary highly anatomically across healthy individuals in gyral and sulcal shape and patterning with a convoluted cortical ribbon. Characteristic signs of TLE are an increased folding complexity in temporo‐limbic cortices, hippocampal malrotations, and developmental anomalies (Kim et al., 2012; Voets, Bernhardt, Kim, Yoon, & Bernasconi, 2011) in addition to disease progression‐related atrophy (Roggenhofer et al., 2019). Individual anatomical particularities and disease‐dependent remodeling of medial temporal lobes can influence the spatial distribution of an affected structural pattern on the group level. In analogy, it was not possible to determine mesial temporal lobe structures as the most affected regions concerning structural differences between TLE and healthy volunteers in the VBM approach.

A multivariate approach can reveal information jointly encoded by several voxels as the multivariate distance between the two categories accounts for correlations among these. Extending classical inferences of mass‐univariate analysis, the multivariate technique is particularly suited to quantify local changes in brain morphology and does not depend on a statistical threshold. Using MVB, we were able to answer the question which brain regions are jointly informative for the disease pathology and which anatomical structures allow to separate TLE patients and healthy controls. In this respect, the established multivariate method provides a reliable and robust ranking.

### 4.4 Limitations and conclusion

We acknowledge several limitations in the design which may have impacted our results. First, we performed a population‐based study whereupon the explanatory power concerning a spatial pattern is limited on the level of the individual patient. With respect to intersubject spatial variability of the epileptogenic focus in TLE, a translation of the method to the individual level provides the possibility to localize the morphological focus in MRI− TLE. The focus detection of structural abnormalities in patients without an EEG‐detectable seizure onset zone can provide a decisive feature within a multimodal presurgical diagnostic framework in drug‐resistant patients. Second, the MVB‐based ranking was paralleled by a VBM‐based ranking, which does not provide a validation of the MVB method. Up to now, it remains unknown to what extent a morphological focus is congruent with the seizure onset zone. To validate the accuracy of the multivariate technique, one could assess the overlap between individual seizure onset zones based on concomitant intracranial EEG recordings and MVB‐based detection of the focus which contributes the most to dynamic brain remodeling.

We present topology inferences of disease‐related remodeling that highlights a key dissociation in the brain anatomy contributing to epilepsy pathology and healthy control conditions. Structural Bayesian modeling furnishes an appropriate framework for population‐based analysis to identify regions being most important for disease‐related structural remodeling and quantitatively estimate the involvement of distinct structures in spatially restricted morphological remodeling in focal epilepsy. An individualized focus identification can provide preoperative clinical benefits for targeting electrodes used for neurostimulation therapy and is relevant to guide and monitor surgical intervention, especially in the context of increased use of minimally invasive approaches, such as MRI‐guided thermal ablation.

## CONFLICT OF INTEREST

Nothing to report.

## AUTHOR CONTRIBUTIONS

E.R., S.M., L.M., R.W., C.R., and B.D. were involved in conception of the project. E.R., S.M., and B.D. designed the study. E.S. and G.V. were involved in acquisition of data. L.M. and E.R. performed imaging preprocessing. E.R. analyzed the data. S.M. translated the MVB approach suited for application in functional MRI to structural MRI. E.R., F.K., and B.D. interpreted the data. E.R. and B.D. prepared the manuscript. All authors reviewed, edited the manuscript and were involved in subsequent revisions.

### Peer Review

The peer review history for this article is available at https://publons.com/publon/10.1002/brb3.1825.

## Data Availability

Data available on request due to privacy/ethical restrictions.
